# Revisiting the *J* = 1 ← 0 fundamental rotational transition of HHe^+^ with action spectroscopy

**DOI:** 10.1039/d5cp04689k

**Published:** 2026-01-27

**Authors:** Oskar Asvany, Urs U. Graf, Weslley G. D. P. Silva, Lea Schneider, Slawa Kabanovic, Volker Ossenkopf-Okada, Jürgen Stutzki, Igor Savić, Rolf Güsten, Oliver Ricken, Bernd Klein, Stephan Schlemmer

**Affiliations:** a I. Physikalisches Institut, Universität zu Köln Zülpicher Str. 77 D-50937 Köln Germany asvany@ph1.uni-koeln.de; b University of Novi Sad, Faculty of Sciences, Department of Physics Novi Sad 21000 Serbia; c Max-Planck-Institut für Radioastronomie Auf dem Hügel 69 53121 Bonn Germany

## Abstract

The *J* = 1 ← 0 fundamental rotational transition of HHe^+^ at 2.010 THz has been revisited using a combination of a 4 K 22-pole ion trap apparatus and a high-power frequency multiplied THz source. For the detection of the resonant absorption, three different action spectroscopic techniques have been applied, one of which is demonstrated here for the first time (ejection of the ion upon pure rotational excitation). The different methods are evaluated and compared, and improve the accuracy and precision of the former transition value by one order of magnitude to 2010.183312(8) GHz.

## Abstract 2

The *J* = 1 ← 0 fundamental rotational transition of HHe^+^ at 2.010 THz has been revisited using a combination of a 4 K 22-pole ion trap apparatus and a high-power frequency multiplied THz source.

## Graphical abstract

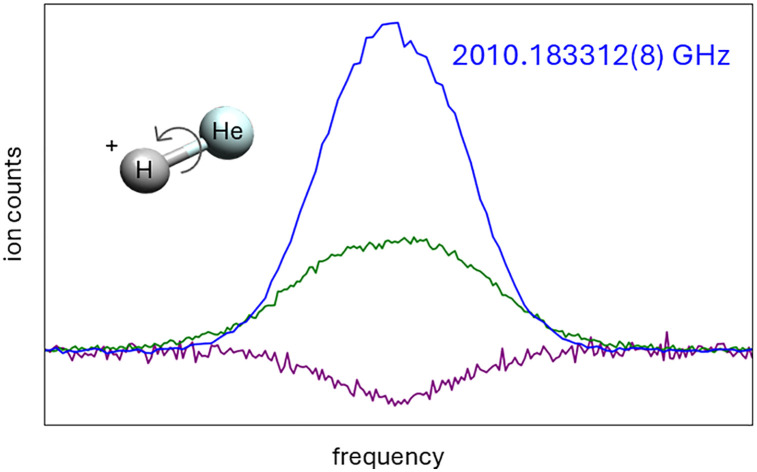

## 1 Introduction

The HHe^+^ cation is a fundamental two-electron system, very similar to the hydrogen molecule H_2_, and is thought to be the very first diatomic molecule formed when the early universe cooled down.^1^ It has been discovered 1925 in a laboratory mass spectrometer by Hogness and Lunn,^2^ and many decades later investigated by high-resolution vibrational^3–6^ and rotational^7–9^ spectroscopy. In particular the direct measurement of the fundamental rotational transition *J* = 1 ← 0 at 2010.1839(2) GHz by Matsushima *et al.*^7^ laid the foundation for the later astrophysical detection of HHe^+^ in the planetary nebula NGC 7027,^10^ achieved with the upGREAT (German Receiver for Astronomy at Terahertz Frequencies) receiver^11^ on-board the SOFIA (Stratospheric Observatory for Infrared Astronomy) airborne observatory.^12^

In this work, we combine a cryogenic ion trapping apparatus with a tunable 2 THz source for high-resolution spectroscopy purposes, the THz source being the very same as used as local oscillator (LO) in the upGREAT receiver mentioned above. In order to evaluate the performance of this setup, we revisit the *J* = 1 ← 0 transition of HHe^+^ with three different action spectroscopic techniques, leading finally to a refinement of the transition frequency value by one order of magnitude.

## 2. Experiment

### 2.1. Cryogenic ion trap machine

The experiments of this study have been carried out in the cryogenic 22-pole ion trapping instrument COLTRAP.^13^ Every second, a pulse of several ten thousand of HHe^+^ ions was generated in a storage ion source by electron impact ionization (electron energy about 45 eV) of a H_2_–He mixture, selected in a quadrupole mass spectrometer for mass range 2–6 u (exact selection was not considered necessary), and then injected into the cryogenic 22-pole ion trap.^14^ The trap was held at a temperature of *T* = 4 K and was filled with inert gases as collision partners (details see below). During the trapping time of about 200 ms, the stored HHe^+^ ion cloud was irradiated with the THz beam. The outcome of this interaction has been probed *via* action spectroscopy (see below) by releasing the ion cloud into a second quadrupole mass spectrometer and counting the ions of interest in a high-efficiency ion counter. By counting the ions as a function of the THz frequency, the rotational transition of HHe^+^ could be recorded.

### 2.2. THz source

The radiation source is the local oscillator (LO) which was utilized for the low frequency array (LFA) of the upGREAT receiver, operating in the range 1.83–2.07 THz.^11^ It contains a sequence of frequency doublers and triplers with a total multiplication factor of 144. For improved efficiency, the last tripler was cooled to about 90 K by a Stirling cooler. In our laboratory, the chain was driven by a Rohde & Schwarz model SMF 100A synthesizer locked to a SRS model FS725 rubidium frequency standard. The THz output power is on the order 40 µW and had to be attenuated by sheets of paper (up to 4 of 80 g m^−2^) for some of our high-sensitivity experiments in order to avoid power saturation. The THz beam reached the 22-pole ion trap *via* a plane 25 mm diameter aluminum mirror and a 0.6 mm thick diamond window (Diamond Materials GmbH). The output beam waist (*w* = 3.25 mm) was located near the position of the trap. For the double resonance experiments described below, a 1 mm hole in another plane mirror was used to overlay the THz radiation with the infrared (IR) beam of an optical parametric oscillator (Toptica, TOPO) operating in the 3 µm region. A schematic view of the setup is given in Fig. S1 of the SI.

### 2.3. Action spectroscopic methods

**Fig. 1:**
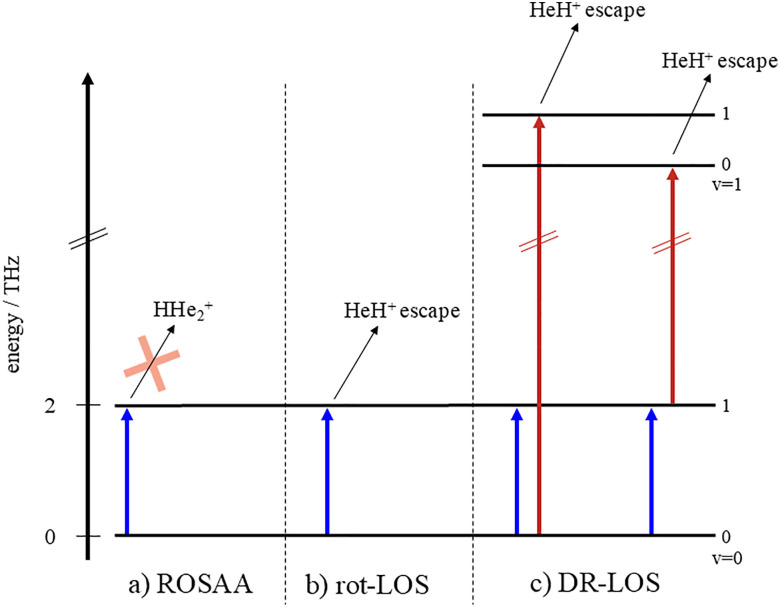
Excitation schemes for the three different action spectroscopic methods used: (a) ROSAA: ROtational State-dependent Attachment of He Atoms. Irradiation with a THz source leads to the reduction of ternary He-attachment. Thus, we observe a dip in the HHe_2_^+^ ion counts (mass 9 u) (b) rot-LOS: rotational leak out spectroscopy. Irradiation with a THz source leads to rotational excitation of the HHe^+^ ions, and subsequent collisions with helium atoms lead to kinetic energies high enough to overcome the trapping potential in the ion trap. The escaping HHe^+^ ions result in an increase of the counts on mass 5 u. (c) DR-LOS: double resonance leak out spectroscopy. Either the THz beam depopulates the state probed by the IR laser, which leads to a dip in the escaping ion count (left), or the THz beam is amplifying the signal generated by LOS, leading to an increase in the HHe^+^ ion count (right).

As direct absorption spectroscopy is prohibited by low number densities in ion trap experiments, action spectroscopy has to be applied. In action spectroscopy, the resonant absorption of the photon is detected *via* its action on the trapped ion ensemble. In our experiments, we applied three different action spectroscopic approaches, which are explained below, and illustrated in Fig. 1.

In the first one (Fig. 1a), the rotational excitation of the molecular ion reduces the three-body rate to attach a He atom to the ion. Therefore, this method is performed by filling the trap with a large number density of He gas (≈10^15^ cm^−3^) for the He-attachment to proceed, and counting the product ions HHe_2_^+^ (mass 9 u) after the trapping period. The resonant transition signal is then detected as a dip in the HHe_2_^+^ counts. This method is very similar to the LIICG method developed for electronic and vibrational excitation (laser/light induced inhibition of complex growth^13,15^) and has been termed ROSAA (ROtational State-dependent Attachment of He Atoms^16^). ROSAA has already been applied to a number of astrophysically relevant cations.^16–25^

With the second action spectroscopic method (Fig. 1b), internally excited ions are kicked out of the ion trap and detected. This is achieved *via* a collision with a neutral atom or molecule, in which the internal energy of the ion is transferred into kinetic energy of both collision partners. For the HHe^+^ measurements, again He gas has been used as collision partner with a relatively low number density (≈10^13^ cm^−3^), and the escaping HHe^+^ ions (mass 5 u) are detected during the trapping/irradiation period. This method has already been demonstrated for rovibrational^26^ and electronic^27^ excitation of the molecular ions and termed leak-out spectroscopy (LOS^26^). In this work, it is demonstrated for the first time that pure rotational excitation can drive the LOS process, and we term the process rot-LOS.

As the third method (Fig. 1c, DR-LOS), a rotational-rovibrational double resonance scheme^28^ based on vibrational LOS has been applied. First, an IR light source is set to the frequency of a known rovibrational transition, creating a steady rovibrational LOS signal. Then, an additional, tunable, THz (or mm-wave) source can excite a ground state rotational transition of HeH^+^, increasing or decreasing the population of the initial state probed by the IR source, thus inducing a change on the LOS signal (a peak or a dip). By repeating the trap cycles (at 1 Hz) and by counting the number of escaping ions as a function of the THz frequency while the IR laser is kept fixed on resonance with the rovibrational transition, the pure rotational line can be recorded. This double-resonance method has also been applied to many cations of astrochemical interest, as HCCCO^+^,^28^ c-C_3_H_2_D^+^,^29^ H_2_CCCH^+^,^30,31^ HCN^+^,^32^ HCNH^+^,^33^ C_3_H^+^,^34^ and NCCO^+^.^35^

## 3. Results

**Fig. 2:**
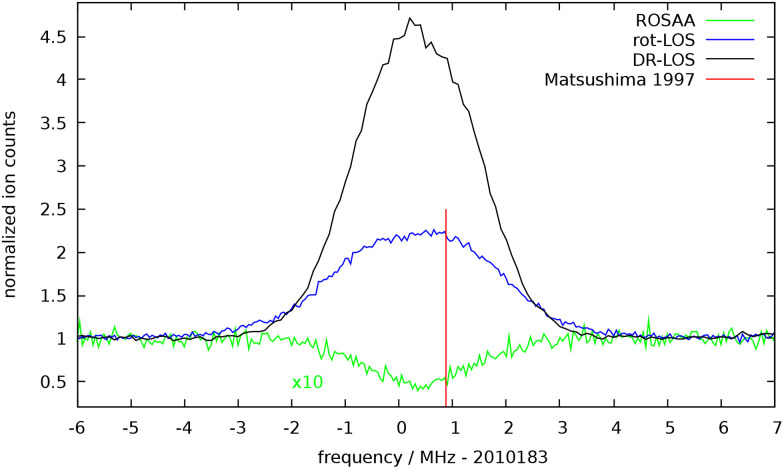
The *J* = 1 ← 0 fundamental rotational transition of HHe^+^, measured with three different action spectroscopic methods: (a) ROSAA: We observe a dip in the HHe_2_^+^ ion counts (mass 9 u) of about 6%. For better visibility, this dip has been magnified by a factor of 10. (b) rot-LOS: the peak corresponds to an increase of the parent ions HHe^+^ of about 100%. This experiment has been conducted with a He number density of about 6 × 10^13^ cm^−3^ in the trap (corresponding to a pressure of main chamber 10^−6^ mbar). (c) DR-LOS: the peak corresponds to an increase of the parent ions HHe^+^ of about 350%. The FWHM of the different measurements is on the order of 3 MHz, corresponding to ion temperatures between 17 and 27 K.

The measured fundamental rotational transition *J* = 1 ← 0 of HHe^+^, obtained with the three action spectroscopic methods, is shown in Fig. 2. For all these measurements, the THz frequency was repeatedly scanned back and forth across the given frequency window, with a typical step size of 50 or 25 kHz. The spectroscopic data were normalized employing a frequency switching procedure, *i.e.*, by dividing the ion counts monitored while scanning the spectral window of interest by the counts at an off-resonant THz reference frequency. Therefore, the baselines in Fig. 2 are close to unity. After this normalization, the signals were averaged for each given frequency position in the scan. The widths of the signals shown in Fig. 2 are dominated by the temperature of the ions (Doppler broadening) and partially also some power broadening effects. When the power was sufficiently attenuated, *e.g.* for the sensitive DR-LOS measurements, we obtain a width corresponding to a temperature of the HHe^+^ ion of about 17 K. This temperature is elevated w.r.t. the nominal trap temperature of 4 K due to known radio-frequency heating effects.^36^

We started our investigations with the well-proven ROSAA method, and after mirror optimization the signal shown in Fig. 2 was obtained. That signal is the accumulated and averaged data of about 14 h of measurement. The normalized signal exhibits a depletion of 6%, and a Gaussian fit to the data yields a center frequency of 2010.18336(3) GHz (the uncertainties given in brackets in this paper correspond to one sigma).

**Fig. 3:**
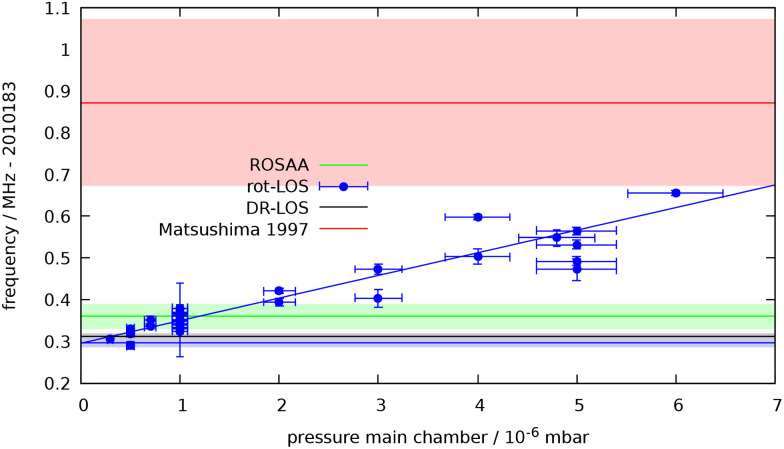
Center frequencies and uncertainties for the *J* = 1 ← 0 fundamental rotational transition of HHe^+^, determined with the three different methods in this work, and comparison to the former result of Matsushima *et al.*^7^ For the rot-LOS method we observe a slight dependence on the He number density (see text), converging to the assumed center frequency at low density. Such a density dependence was tested, but considered negligible for the DR-LOS method (see Fig. S3).

Of particular interest is the rot-LOS method, as it is demonstrated here for the first time. The rotational-to-translational energy transfer is generally assumed to proceed near collision rate thus making the rot-LOS method very efficient. Nonetheless, the good signal-to-noise ratio shown in Fig. 2 is astonishing, as only a very small energy is given to the escaping HHe^+^ ions, which we estimate to be a maximum of 4/(4 + 5)·67.05 cm^−1^ = 29.8 cm^−1^ ≈3.69 meV. This corresponds to a temperature increase of only about 42 K, and is apparently sufficient to allow the ions stored at 17 K to escape. But the ions hardly escape the trap, and are thus susceptible to any collisional interference with the He buffer gas. Indeed, we observe a diminishing rot-LOS signal with increasing He number density in the ion trap (see Fig. S2 in the SI), and an accompanying shift of the fitted peak center to higher frequencies, as shown in Fig. 3. The decrease of the signal for higher pressures hints towards a competition between the rotation to translation kick-out process and secondary (translation–translation) cooling collisions. A similar behavior was found for the vibration to translation process.^37^ The shift of the peak center to higher frequencies can be explained by the fact that the 17 K ions moving initially towards the detector, which in our setup are represented by the higher frequency wing of the Doppler distribution, have a higher probability to reach the detector. These findings are supported by an estimation for the mean free path, *Λ*, of the HHe^+^ ions in the trap at the given He number densities (a pressure of 10^−6^ mbar in the main experimental chamber corresponds to a number density of about *n* = 6 × 10^13^ cm^−3^ inside the trap), which is *Λ* = 0.28 cm and thus much shorter than the trap length (4 cm)††The mean free path is *Λ* = *v*/(*nk*), and we assumed *v* = 316 m s^−1^ and *k* = 1.85 × 10^−9^ cm^3^ s^−1^.. Additionally, we could prove the feasibility of the rot-LOS method also for the *N* = 2 ← 1 transitions of the radical OH^+^ scattered around 2 THz (see also Section 4), and also here we observed a similar diminishing rot-LOS signal with increasing He number density. In summary, extrapolating the pressure dependence in Fig. 3 to zero pressure by a simple linear fit, we obtain a center frequency of 2010.183296(10) GHz for the rot-LOS method, which is in reasonable agreement with our other measurement results.

Finally, we tested the DR-LOS method, using the IR transitions (red arrows in Fig. 1) *P*(1), *R*(1) and *R*(0), whose wavenumbers are tabulated in ref. 5 and 6. Of all applied action spectroscopic methods, the DR-LOS “peak” method (the right-hand scheme in Fig. 1c) is the one with the best S/N ratio (see Fig. 2), due to the high IR photon energy (0.33 eV) driving the LOS process and the constructive order of the THz and IR photons. In contrast, the DR-LOS “dip” method (the left-hand scheme in Fig. 1c) arranges the two photons in competing order, leading to a much poorer S/N ratio (therefore the measurement is not depicted in Fig. 2). We thus used the “peak” DR-LOS method for the final determination of the transition frequency. After combining the frequencies and uncertainties of about 30 such measurements, each consisting of at least 4 up-and-down scans, we arrive at a final transition frequency value of 2010.183312(8) GHz. The low temperature of the current experiment and the good S/N ratio enables this value to be more accurate and precise than given by Matsushima *et al.*^7^ by more than one order of magnitude. A comparison of all frequencies and uncertainties is given in Fig. 3.

## 4. Additional measurements

With the experimental setup and action spectroscopic methods presented in this paper, several other spectroscopic systems can be targeted, the only requirements being (i) the species to be an ion, (ii) its transition originating from a low-lying quantum level, and (iii) being in the range 1.83–2.07 THz. These requirements are met, for instance, by the rotational transitions 3_30_ ← 2_11_ and 4_22_ ← 3_03_ of CH_2_D^+^, the *N* = 2 ← 1 spin manifold of OH^+^, as well as the fine structure transition ^2^P_3/2_ ← ^2^P_1/2_ of the ionized carbon atom, C^+^, at 1.9 THz. Whereas the spectroscopy of OH^+^ using the DR-LOS, ROSAA, and even the rot-LOS methods was successful, and will be presented in detail in a forthcoming publication, our ^12^C^+^ measurements applying LIICG and LOS (THz-only) did not yield any useful signal.

Nevertheless, due to its outstanding astronomical importance, a few words are warranted about the 1.9 THz ^2^P_3/2_ ← ^2^P_1/2_ measurements. This transition, detected for the first time in the interstellar medium by Russell *et al.*,^38^ is known to be one of the most important cooling lines of the interstellar medium and is therefore an important diagnostic of, *e.g.*, star forming activity.^39–45^ Of particular importance are also the corresponding ^13^C^+^ lines (due to hyperfine interaction these split into 3 components) as these lines are optically thin and allow for an accurate column density determination. All recent detections still rely on the laboratory measurements from Cooksy *et al.*,^46^ performed in 1986 with laser magnetic resonance (LMR), where a strong magnetic field is used to shift the Zeeman components of the transition into resonance with fixed molecular laser lines. As only a limited number of Zeeman components could be measured in a high magnetic field by the LMR method, with only 2 lines measured for the isotope ^13^C^+^, the determination of the zero-field hyperfine frequency components of ^13^C^+^ had to rely on *ab initio* calculations.

It therefore seems highly desirable to revisit the hyperfine components of this fine structure transition with our LIICG and LOS methods. The LOS process itself should be highly feasible, as theoretical calculations^47–49^ yield a quenching rate coefficient for the astrochemically important collisions of C^+^(^2^P_3/2_) + He and C^+^(^2^P_3/2_) + H_2_ which are a substantial fraction of the Langevin collision rate coefficient, and the quenching rates for collisions with the partners Ne and N_2_ (O_2_ cannot be taken as it is reactive) as used in this work should be only smaller by a factor of a few (priv. communication François Lique and Alexandre Faure). But unfortunately, the electric dipole-forbidden ^2^P_3/2_ ← ^2^P_1/2_ transition has an Einstein coefficient *A* = 2.29 × 10^−6^ s^−1^ (see ref. 50) and is thus more than 4 orders of magnitude smaller than the Einstein coefficient *A* = 0.087 s^−1^ (taken from CDMS repository^51^) of the *J* = 1 ← 0 fundamental rotational transition of HHe^+^ investigated in this work. For C^+^, we thus calculate the Einstein coefficient for excitation *B* = 1.62 × 10^15^ m^3^ J s^−2^, and with 40 µW power we estimate the excitation rate of the fine structure transition to be on the order of only 1.3 s^−1^. We thus conclude that a 1.9 THz source with higher power will be needed to reinvestigate this transition in future.

## 5. Discussion of HHe^+^ results

Our transition frequency value is about 560 kHz lower than that given by Matsushima *et al.*,^7^ which corresponds to about 2.8 times their uncertainty (200 kHz), see Fig. 3. This can be considered a fair agreement, but nevertheless we checked our frequency calibration by using two different rubidium clocks driving our synthesizer. An additional hint for a somewhat lower transition value as given by Matsushima *et al.*^7^ can be found by performing a global fit. By including all available high-resolution data,^5–8^ except the transition under consideration, we indeed obtain a lower predicted transition value of 2010.18337(49) GHz which is close to our new value. Including our improved value into this global fit, we can refine the rotational constant *B*_0_ by a factor of about 3–4 compared to its most recent published value.^6^ The new fit results will eventually appear on the CDMS^51^ repository.

The astronomical results reported by Güsten *et al.*^10^ are not affected by the 0.08 km s^−1^ shift in Doppler velocity imposed by the new rest frequency value. But the newly determined transition frequency may serve as a strict benchmark value for future *ab initio* calculations of the fundamental two-nucleus-two-electron system HHe^+^. Such calculations typically take into account adiabatic and nonadiabatic corrections as well as relativistic and quantum electrodynamics effects,^52,53^ and some even go beyond the Born–Oppenheimer approximation.^54,55^ While calculations from the year 2012^52,53^ had a predictive power on the order of 0.001 cm^−1^ = 30 MHz for the *J* = 1 ← 0 transition of HHe^+^, the most recent *ab initio* values from the Poznań group,^56,57^ of which we got to know during the review of this paper, made a tremendous step towards spectroscopic accuracy, with a transition frequency of 2010183.229(90) MHz. This value gives mutual confirmation of our and their results. An overview of all experimental and computed transition values mentioned in this work is given in Table S1 of the SI.

The systematic shift of the peak center frequency with trap pressure (number density) for the rot-LOS method is a surprising but consistent result which seems to be associated with the velocity group selected by the excitation frequency. This effect has not been found for the DR-LOS method, as the kick-out process there is governed by the less probable V–T-transfer process which does not seem to favor a velocity group. This is a good indication that the transition frequencies found by DR-LOS in all our previous investigations are rather accurate. Nevertheless, more systematic line-shape and line-shift studies are required to explore the limits of frequency accuracy using leak-out spectroscopy and to understand the underlying collision processes in more detail.

Finally, for the HHe^+^ + He collision leading to the LOS signal, we assumed the rotational or vibrational excitation of the ion to be transferred to both collision partners which stay intact during collision. But in this special case, we cannot rule out a proton-hop to occur and lead to the same signal. Future experiments, in which one of the collision partners is ^3^He-substituted, will give a clearer answer, and it will be interesting to investigate this separately for rotational excitation (rot-LOS) and rovibrational excitation (DR-LOS).

## Conflicts of interest

There are no conflicts to declare.

## Supplementary Material

CP-028-D5CP04689K-s001

## Data Availability

All the important data (one transition frequency) is contained in the text of the submitted work. Supplementary information (SI) is available. See DOI: https://doi.org/10.1039/d5cp04689k.
