# Supplementary material for: Revisiting the J = 1 ← 0 fundamental rotational transition of HHe+ with action spectroscopy
Source: Phys Chem Chem Phys. 2026 Jan 27;28(7):4662–7. doi: 10.1039/d5cp04689k (PMC12865889; doi:10.1039/d5cp04689k)
Supplement: CP-028-D5CP04689K-s001 [file CP-028-D5CP04689K-s001.pdf]

Supplementary Information (SI) for Physical Chemistry Chemical Physics.
This journal is © the Owner Societies 2026

Electronic Supporting Information: Revisiting the $J = 1 \leftarrow 0$ fundamental rotational transition of HHe^+ with action spectroscopy

Oskar Asvany [a],* Urs U. Graf [a], Weslley G. D. P. Silva [a], Lea Schneider [a], Slawa Kabanovic [a], Volker Ossenkopf [a], Jürgen Stutzki [a], Igor Savić [b], Rolf Güsten [c], Oliver Ricken [c], Bernd Klein [c], and Stephan Schlemmer [a]

[a] *I. Physikalisches Institut, Universität zu Köln, Zülpicher Str. 77, 50937 Köln, Germany.*

[b] *University of Novi Sad, Department of Physics, Novi Sad 21000, Serbia*

[c] *Max-Planck-Institut für Radioastronomie, Auf dem Hügel 69, 53121 Bonn, Germany*

E-mail: asvany@ph1.uni-koeln.de

Overview of experimental setup

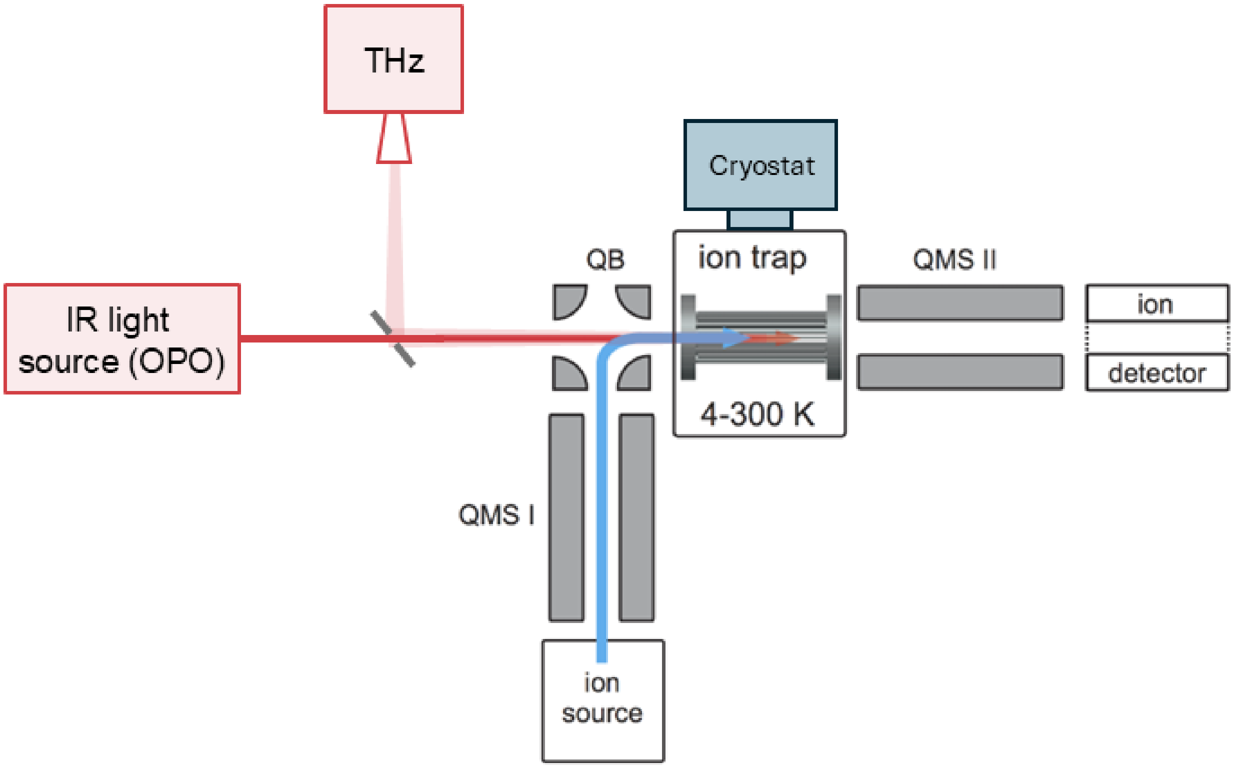

Figure S1: Schematic setup of the ion trapping apparatus (QMS: Quadrupole mass spectrometer, QB: quadrupole bender). The HHe^+ ions are generated in the ion source, mass selected in QMS I, and then trapped in the ion trap at 4 K. During the trapping time, they are irradiated with narrow-band radiation. For the ROSAA and rot-LOS methods, only the 2 THz source is used, while for the DR-LOS method a high-resolution OPO is used in addition. A small hole in the flat mirror superimposes the two beams.

Dependence of rot-LOS and DR-LOS signal on helium density in ion trap

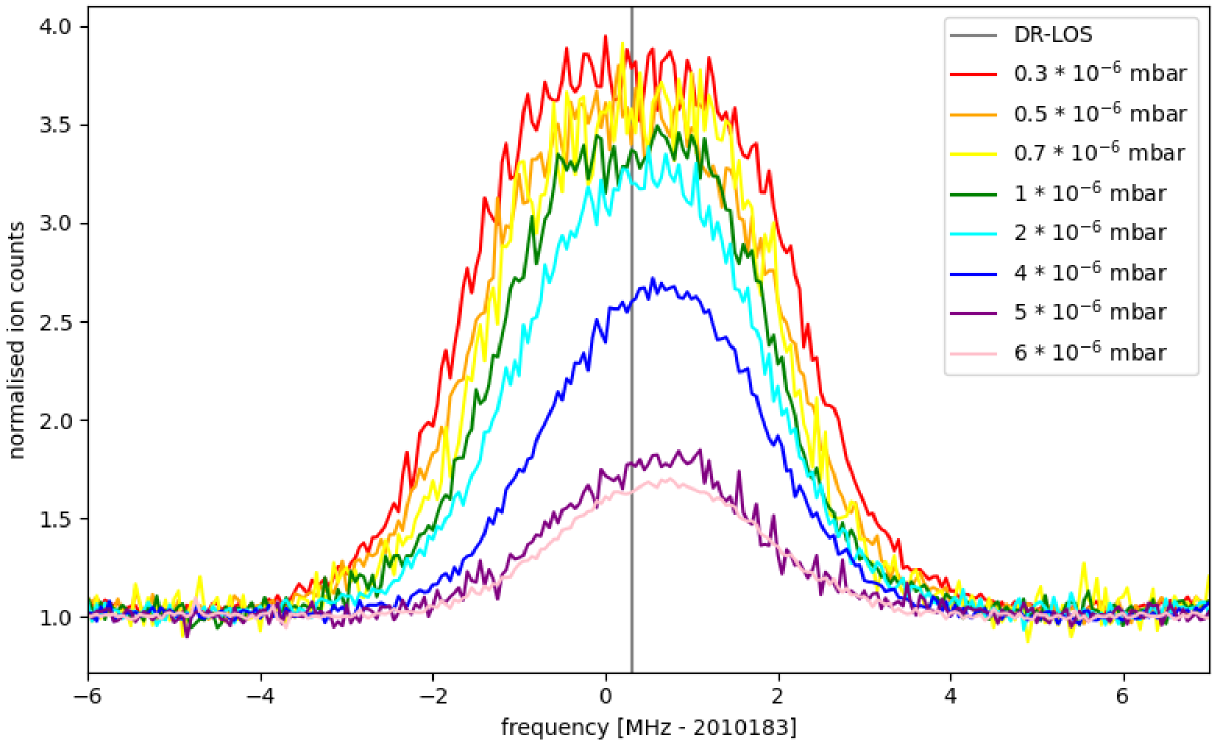

Figure S2: Several rot-LOS measurements of the $J = 1 \leftarrow 0$ rotational transition of HHe^+ as a function of the helium density in the ion trap. The pressure given is that of the ion gauge of the main vacuum chamber which is proportional to the helium density in the trap. With increasing He density, the signal is diminishing and shifting to higher frequencies. We interpret this as being caused by secondary collisions with the He buffer gas, which hinders the low-energy HHe^+ ions from leaving the trap. More details are given in the main article.

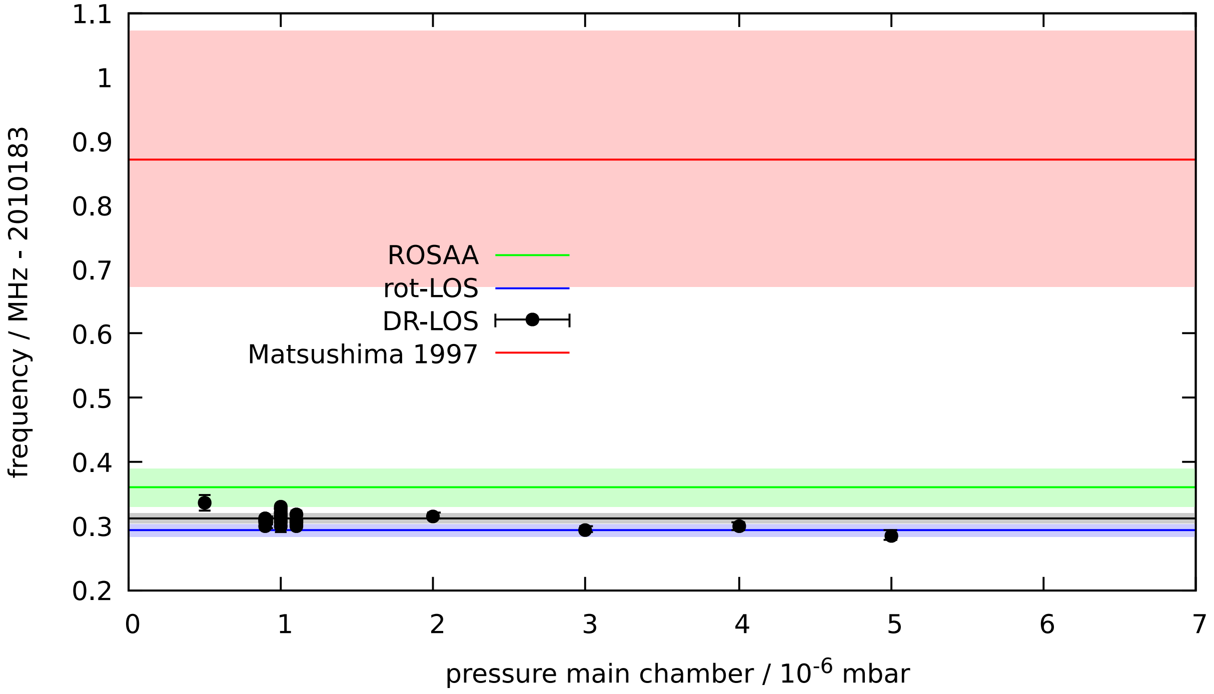

Figure S3: This Figure is the same as Fig. 3 of the main manuscript, but it shows in more detail the He pressure dependence of the 31 independent DR-LOS measurements (black dots with errorbars). The majority of these measurements (26 measurements) have been done at a pressure of $0.9 - 1.1 \times 10^{-6}$ mbar (ion gauge reading of the main vacuum chamber), and only 5 measurements at higher and lower pressure. The eye might be guided to see a negative pressure dependence, but which is hardly statistically significant (about -5.6 ± 3.5 kHz/10^{-6} mbar). Due to the limited dataset at higher pressures it is unclear whether this tendency is due to statistical error, a systematic shift of the measurement method, or the onset of a classical pressure shift. The transition frequency of the $J = 1 \leftarrow 0$ rotational transition of HHe^+ as given in the main manuscript is untouched by these findings.

Table S1: Comparison of the frequency values for the $J = 1 \leftarrow 0$ fundamental rotational transition of HeH^+ (in MHz), obtained in this and other works.

Frequency	exp/theo	reference
2010183.9(2)	exp	Matsushima et al 1997
2010183.36(3)	exp	this work, ROSAA
2010183.296(10)	exp	this work, rot-LOS
2010183.312(8)	exp	this work, DR-LOS
2010183.229(90)	*ab initio*	Siłkowski et al 2026
